# Improved performance of cementless total knee arthroplasty (TKA)across international registries: a comparative review

**DOI:** 10.1007/s11845-025-03888-6

**Published:** 2025-02-10

**Authors:** Meadhbh Ni Mhiochain de Grae, Armon Nasehi, David F. Dalury, Bas A. Masri, Gerard A. Sheridan

**Affiliations:** 1 Department of Orthopaedic Surgery, University of Galway, Galway, Ireland; 2 St. Joseph Medical Center, Baltimore, U.S.A.; 3 Department of Orthopaedics, University of British Columbia, Vancouver, BC Canada

**Keywords:** Cementless, Registry, Revision, TKA, TKR

## Abstract

**Background:**

Cementless fixation in total knee arthroplasty (TKA) has been associated with higher revision rates in the past. However, due to advancements in design, as well as surgical techniques, cementless TKA performance has significantly improved. The advantages of cementless fixation include reduced cement-related complications, shorter operating times, and the potential benefits of osseointegration. We aim to assess the improvement in revision rates for cementless TKA over the last 9 years based on international registry reports.

**Methods:**

A comprehensive retrospective review was conducted of six major English-speaking knee arthroplasty registries across the world including the National Joint Registry of England and Wales, Northern Islan Isle of Man and Guernsey (herby referred to as British), Swedish, Canadian, American, Australian, and New Zealand National Joint Registry. Data was collected from the year 2014 along with the most recent annual report published: 2022 or 2023. Data points collected included usage rates of cemented and cementless prostheses for primary TKA, their respective revision rates, and indications for revision.

**Results:**

Across four databases, there was an average 8.3% increase in the utilization of cementless fixation for primary TKA over the past decade. Three registries reported a reduction in revision rates for cementless fixation. Lower revision rates for cementless compared to cemented TKA were observed in the most recent American (3.2% cemented vs. 2.8% cementless) and New Zealand annual reports (11.8% cemented vs. 4.5% cementless). In 2022, the British registry reported lower rates of revision for infection with cementless fixation (0.56 vs. 0.89).

**Conclusion:**

International registries demonstrate increased utilization of cementless TKA. Cementless TKA was reported to have lower revision rates in the most recent US and New Zealand annual reports when compared to cemented TKA.

## Introduction

In 2020, osteoarthritis (OA) ranked as the fourth leading cause of disability [1]. Knee OA is estimated to affect 3.48% of the population, with 25% of individuals affected experiencing life-limiting disability [1, 2]. The United States alone performs over 670,000 primary total knee arthroplasty (TKA) procedures annually [3].

Three primary fixation methods are employed for the components of the knee joint in individuals undergoing TKA: cemented, uncemented, and hybrid fixation [4]. Cemented arthroplasty involves the use of polymethylmethacrylate bone cement to anchor the implant to the bone [5]. Cementless implants have porous metal surfaces which work based on osseointegration [5]. Hybrid fixation involves the use of an uncemented femoral component and a cemented tibial component [6].

Historically, cementless fixation has been linked to higher revision rates compared to cemented fixation, with elderly females experiencing particularly elevated revision rates [7, 8]. Initial iterations of cementless prostheses faced challenges stemming from suboptimal geometry, ineffective osteoconductive surfaces, and insufficient early stable fixation properties [9]. Subsidence is an important consideration, especially in the elderly with poor bone. In select cases, cementation may reduce this risk (i.e., hybrid fixation) but even in desert patients, if the tray is well seated on the cortical rim of the tibial cut surface, subsidence is less likely to occur [10]. Due to cementless fixation relying heavily on the interface fit, there is a concern for a higher risk of periprosthetic fractures [7]. However, recent advancements, including the use of porous coatings, plasma spray, and rotating platforms, have significantly improved outcomes by reducing stress and micromotion at the bone-metal interface [11].

The majority of primary TKAs worldwide (95% in the British and 68.1% in Australia) are now utilizing cemented prostheses [12, 13]. Cemented fixation does have limitations including the risk of bone cement implantation syndrome, aseptic loosening, and extremely challenging revision surgery [7]. Recently, there has been renewed interest in cementless fixation due to advancements in design and manufacturing as well as improvements in surgical technique, reducing the previously reported failure rates [7].

The advantages of cementless fixation encompass a reduction in cement-related complications and shorter operating and tourniquet times [12, 14]. A 2020 meta-analysis revealed no significant disparity in revision rates or post-operative functional knee scores between cemented and cementless implants during a 16.6-year follow-up period [12].

The younger age as well as the higher BMIs of many individuals undergoing total knee arthroplasty (TKA) has promoted consideration for more stable fixation methods that will last the entirety of these young patient’s lifetimes. Cemented fixation is likely inferior to withstand chronic stress on the cement mantle, which cannot remodel compared to a biologically osseointegrated component, potentially leading to higher rates of aseptic loosening [12, 15].

Our study aimed to assess the trends in usage and revision rates for primary cementless total knee arthroplasty (TKA) over the last 9 years based on international registry reports. The hypothesis is that with newer implant designs, survivorship would improve in more recent registry reports.

## Methods

A comprehensive retrospective review was conducted of six major English-speaking knee arthroplasty registries across the world including the British, Canadian, Swedish, American, Australian, and New Zealand National Joint Registry. Data was collected using the online publications of these registries. Data from the year 2014 along with the most recent annual report published; 2022 or 2023 were included. Data points collected included usage rates of cemented and cementless prostheses for primary TKA, their respective revisions rates, and indications for revision if reported.

The inclusion criteria were defined to capture reports of TKA’s both cemented and uncemented. Hybrid prostheses, revision surgeries, and component revision were excluded. We performed a simple descriptive statistical analysis of the collected observational data to elucidate trends, patterns, and associations. Numbers of revisions were converted to percentages to provide uniformity in comparison. Ethics approval was granted by our local ethics committee.

Tables 1, 2, and 3 show the 10-year revision rates for cementless, cemented, and the usage rates of cementless and cemented joints in 2014 and the most recently published registries.

**Table 1:** Comparison of 10-year revision rates across international joint registries (2014 vs. 2022/2023) for cementlessTKA (total knee arthroplasty)

Registry	2014 10-year revision rate	2022/2023 10-year revision rate	Difference in revision rate
National Joint Registry (British)	4.49%	3.89%	⇓0.6%
Australian Registry	6.3%	5.2%	⇓1.1%
New Zealand Registry	5.5%	4.5%	⇓1.0%
Swedish Registry	Not documented	6.5%	NA
Canadian Registry	Not documented	3.62%	NA
American Joint Registry	Not documented	2.8%	NA

**Table 2:** Comparison of 10-year revision rates across international joint registries (2014 vs. 2022/2023) for Cemented TKA (total knee arthroplasty)

Registry	2014 10-year revision rate	2022/2023 10-year revision rate	Difference in revision rate
National Joint Registry (British)	3.33%	2.4%	⇓0.93%
Australian Registry	3.19%	4.0%	⇑ 0.81%
New Zealand Registry	2.7%	11.8%	⇑ 9.1%
Swedish Registry	4%	3%	⇓ 1%
Canadian registry	Not documented	3.46%	NA
American Joint Registry	Not documented	3.2%	NA

**Table 3:** Comparison of rates of cemented and cementless prosthetics used in primary Total Knee Arthroplasty (TKA) in International registries

Registry	Cemented 2014	Cementless 2014	Cemented 2022/2023*	Cementless 2022/2023*	Change in use of cementless	Change in use of cemented
National Joint Registry (British)	93.2%	5.6%	95.4%	3.8%	⇑ 1.8%	⇑ 2.2%
Australian Registry	55%	20%	61.8%	18.3%	⇓ 1.7%	⇑ 6.8%
New Zealand Registry	95%	4%	85%	14%	⇑ 10%	⇓ 10%
Swedish Registry	94.9%	4.7%	90.5%	9.1%	⇑4.4%	⇓ 4.4%
Canadian Registry	89.1%	2.4%	Not documented	Not documented	NA	NA
American Joint Registry	4.1%	2.8%	1.9%	20.5%	⇑ 17.1%	⇓ 2.2%

^*^Most recent registry available reviewed

## Results

### Usage rates of cementless joints

The British National Joint Registry observed a rise of 1.8% in the adoption of cementless implants for primary TKA. Similarly, the New Zealand registry documented a 10% increase, the Swedish registry noted a 4.4% rise, and the American Joint registry showed an increase of 17.1%. Combining data from these four registries reveals an average increase of 8.3% in the usage of cementless fixation for primary TKA over the past decade.

Only the Australian registry recorded a decrease (1.7%) in the adoption of cementless fixation.

On the corollary, the use of cemented fixation has reduced over the past decade in three out of the five registries (Table 3). The New Zealand registry documented a 10% reduction in the use of cemented fixation, the Swedish registry showed a 4.4% reduction, and the US joint registry showed a 2.2% reduction in use. Only the British and the Australian registries showed an increase in cemented fixation use (2.2% and 6.8%, respectively.)

Robotic-assisted surgery has gained popularity in recent years due to its precision, improved alignment, and reproducibility [16]. These advancements may play a role in the preference for cementless fixation, as robotic systems can ensure more accurate bone preparation and implant positioning, which are critical factors for successful cementless fixation [16].

### Revision rates of cementless and cemented joints

As shown in Table 1, all three registries with available trending data reported a reduction in revision rates for cementless fixation since 2014. The British registry reported a 0.6% reduction, the Australian registry documented a 1.1% reduction, and the New Zealand registry showed a 1.0% reduction.

Revision rates for cemented fixation (Table 2) were more heterogenous between registries with the British and Swedish reports showing a reduction in revision rates (0.93% and 1%, respectively), while the Australian and New Zealand showing an increase (0.81% and 9.1%, respectively).

Notably, lower revision rates for cementless compared to cemented TKA are observed in the most recent American (3.2% cemented vs. 2.8% cementless) and New Zealand annual reports (11.8% cemented vs. 4.5% cementless) [17, 18].

### Indications for revision

**Fig. 1:**
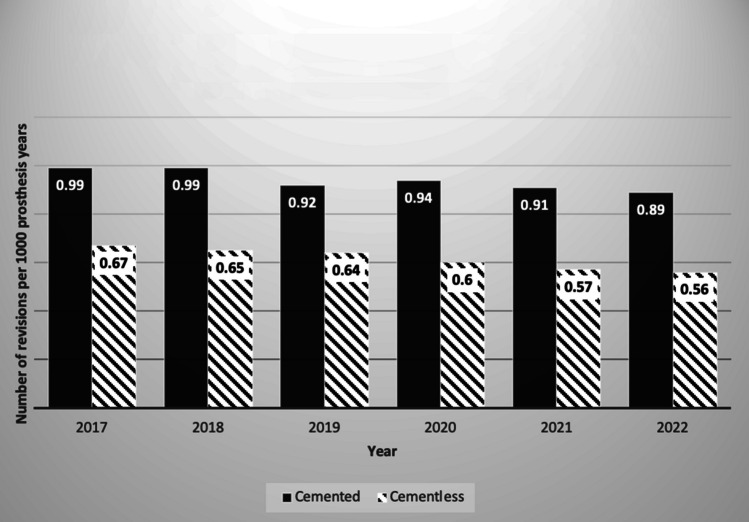
Revision total knee arthroplasties performed due to infection in cemented vs cementless joints annually as per the British National Joint Registry

**Fig. 2:**
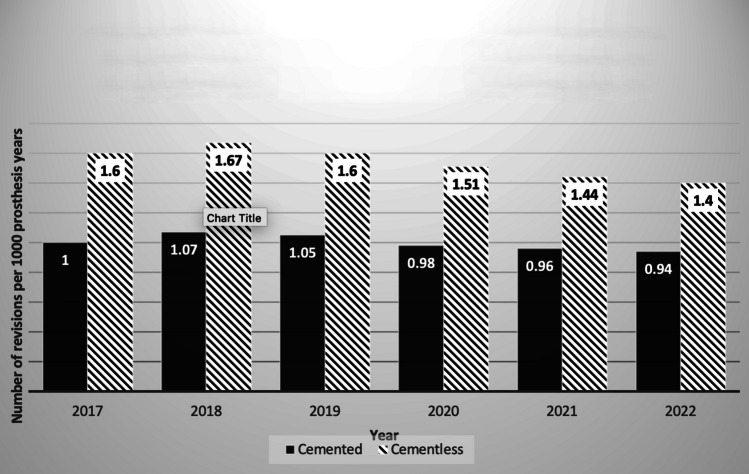
Revision total knee arthroplasties performed due to aseptic loosening in cemented vs cementless joints annually as per the British National Joint Registry

**Fig. 3:**
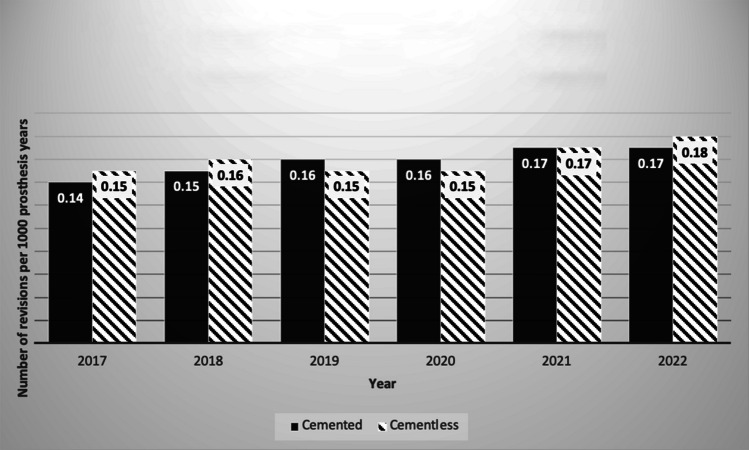
Revision total knee arthroplasty performed due to peri-prosthetic fracture in cemented vs cementless joints annually as per the British National Joint Registry

Only the British registry reported revision rates for specific indications for cemented and cementless TKA between the years 2017 and 2022. These results are displayed graphically in Figs. 1, 2 and 3.

Figure 1 shows a progressive reduction in revision rates for infection with cementless joints, with 0.67 revisions per 1000 prosthesis years in 2017, reducing to 0.56 revisions per 1000 prosthesis years in 2022 [19]. These figures were lower than the respective revision rates for cemented prostheses which were 0.99 revision per 1000 prostheses years in 2017, reducing to 0.89 revision per 1000 in 2022 [19]. Notably, despite the option for antibiotic cement in the cemented cohort, revision rates for infection remain lower in the cementless group.

Figure 2 portrays a decrease in revision rates attributed to aseptic loosening for both cemented and cementless joints. Aseptic loosening rates were higher with cementless fixation in 2022 (0.94 vs. 1.4) [19].

Furthermore, Fig. 3 showcases the revision rates concerning peri-prosthetic fractures, revealing minimal disparity between cemented and cementless prostheses across the analyzed years, with rates of 0.17 per 1000 prosthesis years in cemented vs. 0.18 per 1000 prosthesis years in cementless. Interestingly, rates of revision for peri-prosthetic fracture for both cemented and cementless joints have marginally increased since 2017.

In terms of fixation methods, the British 2022 registry reported the lowest aseptic revision rates for uncemented fixation using constrained but not posterior stabilized and without patella (0.48 revisions per 1000 prosthesis-years). The lowest for cemented was monobloc polyethylene tibia, with the patella (0.57) [19].

## Discussion

Our findings within the international databases show a shift in the utilization of cementless fixation for primary TKA over the last decade. An increase of 8.3% in usage rates over the 10-year period demonstrates renewed interest in this fixation method. Notably, the United States stands out with a significant surge in cementless fixation usage, increasing from 2.8% in 2014 to 20.5% in 2022 [17]. This resurgence in the popularity of cementless fixation may be attributed to conceptual advantages such as enhanced osseointegration facilitating biological fixation, reduced operating times, reduced systemic complications linked to cement impaction and wear from cement debris, and possibly marketing [16].

Revision surgery is associated with poor patient satisfaction, increased complications and added cost [20]. Previously, cementless fixation was associated with higher revision rates. However, both the New Zealand and US registries reported lower revision rates with cementless compared to cemented fixation (see Tables 1 and 2) [17, 18]. The US has shown a consistently lower cumulative revision rate for cementless implants since 2012, deviating from historical trends which showed higher revision rates for cementless TKA [13, 17]. This shift suggests that cementless fixation may offer improved long-term outcomes and durability, potentially reducing the need for costly and invasive revision surgeries. Cementless fixation revision surgery is also associated with less bone loss compared with cemented fixation due to the difficulty of removing cement intra-operatively from the bone surfaces [21].

However, it is essential to examine the underlying reasons behind the revision rate disparities. The theoretical superiority of cemented arthroplasty for reducing periprosthetic joint infection, due to the potential of using antibiotic-infused bone cement (AIBC), has been disproven in multiple reviews [12, 22]. These reviews demonstrated no significant difference in infection rates between cemented and cementless fixation [12]. Interestingly, in 2022, the British registry reported higher rates of revision for infection with cemented fixation (0.89 vs. 0.56) [19]. The US report demonstrated no difference in the risk of revision for infection [17].

A 2017 meta-analysis demonstrated excellent survivorship and functional outcomes with the use of modern cementless TKA with a low incidence of aseptic loosening [23]. Interestingly, aseptic revision rates remained higher with cementless TKA than cemented (0.94 vs. 1.4). However, this rate is reducing for cementless fixation (1.07 revisions per 1000 prosthesis-years in 2018, 0.94 in 2022) as well as cemented fixation (1.67 in 2018 and 1.4 in 2022) [24, 25]. This trend is contradictory to a recent meta-analysis which demonstrated a statistically significantly lower rate of aseptic loosening within the cementless group (OR = 1.62, *P* = 0.02) [26]. We believe that this difference is related to registries having different types of implants, some with a newer design and some without, whereas papers that review specific implants or designs will be limited to these designs. Registry outcomes will lag as all surgeons adopt newer designs and all manufacturers switch to newer implant types.

Periprosthetic fracture (PPF) is a serious complication of knee arthroplasty, often requiring complex revision surgery, but more commonly open reduction and internal fixation [7]. Traditionally, cementless components have been associated with a higher risk of PPF due to their dependency on a close interface fit for stability [7]. However, these studies were based on early designs which had limited ability to contour to the native osseous architecture [16, 27, 28]. Newer designs, materials, and surgical techniques have shown significant improvements [29].

The data available regarding PPF rates is limited as registries only record fractures treated by revision surgery. This is defined as the removal, exchange, or addition of components from the primary operation [7]. Fractures treated with internal fixation, external fixation, and nonoperatively are excluded. A study published in 2023, encompassing all PPFs irrespective of treatment approach, demonstrated no statistically significant difference in incidence rates. The 10-year cumulative fracture rate was 1.2% with cemented components and 1.4% with cementless components [7].

Demographic considerations may also play a role when determining the optimal fixation method. For instance, the 2023 US registry notes that cementless fixation is associated with a reduced rate of cumulative percent revision in all-age men but a significantly increased rate in women aged 65 and older [17]. This may be related to higher rates of osteoporosis in women [30].

Younger age is a risk factor for higher revision rates [31]. There is hope that cementless fixation will be superior to cementless fixation within a younger cohort with higher rates of failure reported with cemented TKA in younger, obese and active patients [32]. Theoretically, the reduced survivorship demonstrated with cemented arthroplasty is due to increased sheer forces and stress at the bone-cement interface, leading to micromotion and aseptic loosening [16, 24]. Cementless fixation overcomes this by promoting biological osseointegration [16]. A 2019 meta-analysis showed vastly superior outcomes for younger patients using cementless fixation including improvement in pain and reduced incidence of aseptic loosening [33].

### Limitations

This was a retrospective study and so all limitations with this study design type apply. The data used is generated from large population sample sizes and so is not available for granular analysis or robust scrutiny. The usage and revision rates are not divided by demographics. For this reason, we decided to only analyze trends and overall rates of utility and revision with cementless TKA. Current revision rates may include a mixture of newer and older generation cementless TKA designs and so the revision rates for cementless TKA implants may be overstated if it were compared to a cohort consisting of exclusively newer generation TKA implants. For this reason, continued cohort studies consisting of only newer-generation cementless implant outcomes are essential.

## Conclusion

Within the international databases, there has been a shift toward the utilization of cementless fixation for primary TKA over the last decade. A surge of 8.3% in usage rates, coupled with lower revision rates compared with cemented prosthesis demonstrated in the New Zealand and US registries, shows the value of cementless fixation as well as highlighting its potential to surpass cemented fixation as the preferred option for primary TKA in the future.

## References

[1] Mahir L, Belhaj K, Zahi S et al (2016) Impact of knee osteoarthritis on the quality of life. Ann Phys Rehabil Med 59:e159

[2] Cross M, Smith E, Hoy D et al (2014) The global burden of hip and knee osteoarthritis: estimates from the global burden of disease 2010 study. Ann Rheum Dis 73(7):1323–1330 24553908 10.1136/annrheumdis-2013-204763

[3] Skou ST, Roos EM, Laursen MB et al (2015) A randomized, controlled trial of total knee replacement. N Engl J Med 373(17):1597–1606 26488691 10.1056/NEJMoa1505467

[4] Fozo ZA, Hussein Ghazal A, Kamal I et al (2023) A systematic review and network meta-analysis of the outcomes of patients with total knee arthroplasty using cemented, uncemented, or hybrid techniques. Cureus 15(10):e47299 37869049 10.7759/cureus.47299 PMC10589057

[5] Bauer TW, Schils J (1999) The pathology of total joint arthroplasty: I. Mechanisms of implant fixation. Skeletal Radiol 28:423–432 10486010 10.1007/s002560050541

[6] McLaughlin JR, Lee KR (2014) Hybrid total knee arthroplasty: 10-to 16-year follow-up. Orthopedics 37(11):e975–e977 25361373 10.3928/01477447-20141023-53

[7] Mohammad HR, Judge A, Murray DW (2023) A comparison of the periprosthetic fracture rate of cemented and cementless total knee arthroplasties: an analysis of data from the National Joint Registry. J Arthroplast 39(6):1505-11 10.1016/j.arth.2023.11.039 38056722

[8] Mosher ZA, Bolognesi MP, Malkani AL et al (2024) Cementless total knee arthroplasty: a resurgence—who, when, where, and how? J Arthroplasty 39(9):S45–S53 38458333 10.1016/j.arth.2024.02.078

[9] Chiou D, Li AK, Upfill-Brown A et al (2023) Cementless compared to cemented total knee arthroplasty is associated with more revisions within 1 year of index surgery. Arthroplasty Today 21 10.1016/j.artd.2023.101122 PMC10382689 37521088

[10] Gibbons JP, Cassidy RS, Bryce L et al (2023) Is cementless total knee arthroplasty safe in women over 75 Y of age? J Arthroplasty 38(4):691–699 36272510 10.1016/j.arth.2022.10.021

[11] Aprato A, Risitano S, Sabatini L et al (2016) Cementless total knee arthroplasty. Ann Transl Med 4(7):129 27162779 10.21037/atm.2016.01.34 PMC4842397

[12] Prasad AK, Tan JH, Bedair HS et al (2020) Cemented vs. cementless fixation in primary total knee arthroplasty: a systematic review and meta-analysis. EFORT open reviews 5(11):793–798 10.1302/2058-5241.5.200030 PMC7722941 33312706

[13] Gioe TJ, Novak C, Sinner P et al (2007) Knee arthroplasty in the young patient: survival in a community registry. Clin Orthop Relat Res 464:83–87 17589362 10.1097/BLO.0b013e31812f79a9

[14] Sheridan GA, Cassidy RS, McKee C et al (2023) Survivorship of 500 cementless total knee arthroplasties in patients under 55 years of age. J Arthroplasty 38(5):820–823 36309144 10.1016/j.arth.2022.10.035

[15] Miller MA, Goodheart JR, Izant TH et al (2014) Loss of cement-bone interlock in retrieved tibial components from total knee arthroplasties. Clin Orthop Relat Res® 472(1):304–313 10.1007/s11999-013-3248-4 PMC3889460 23975251

[16] Asokan A, Plastow R, Kayani B et al (2021) Cementless knee arthroplasty: a review of recent performance. Bone & Joint Open 2(1):48–57 33537676 10.1302/2633-1462.21.BJO-2020-0172.R1 PMC7842161

[17] American Academy of Orthoapedic Surgeons (2023) AJRR 2023 annual report, American Academy of Orthoapedic Surgeons. Available at: https://connect.registryapps.net/2023-ajrr-annual-report. Accessed 30 Jan 2025

[18] (NZOA) NZOA (2023) The New Zealand Joint Registry twenty-four year report January 1999 to December 2022. New Zealand Joint Registry

[19] Ben-Shlomo Y, Blom A, Boulton C et al (2022) National Joint Registry Annual Reports. The National Joint Registry 19th Annual Report 2022. National Joint Registry, London 36516281

[20] Jorgensen NB, McAuliffe M, Orschulok T et al (2019) Major aseptic revision following total knee replacement: a study of 478,081 total knee replacements from the Australian Orthopaedic Association National Joint Replacement Registry. JBJS 101(4):302–310 10.2106/JBJS.17.01528 30801369

[21] Patel AR, Barlow B, Ranawat AS (2015) Stem length in revision total knee arthroplasty. Curr Rev Musculoskelet Med 8:407–412 26371072 10.1007/s12178-015-9297-4 PMC4630236

[22] Belt HVD, Neut D, Schenk W et al (2001) Infection of orthopaedic implants and the use of antibiotic-loaded bone cements: a review. Acta Orthopaedica Scandinavica 72(6):557–571 11817870 10.1080/000164701317268978

[23] van der List JP, Sheng DL, Kleeblad LJ et al (2017) Outcomes of cementless unicompartmental and total knee arthroplasty: a systematic review. Knee 24(3):497–507 27923627 10.1016/j.knee.2016.10.010

[24] NJR 20th Annual Report 2023 Available at: https://reports.njrcentr.org.ul/Portals/0/PDFdownloads/NJR%202th%20Annual%20Report%202023.pdf. Accessed: 30 Jan 2025

[25] National Joint Registry for England, Wales and Northern Ireland 11th Annual Report 2024 Available at: https://reports.njrcentre.org.uk/Portals/1/PDF.downloads/NJR%2011th%20Annual%20Report%202014.pdf. Accessed 3 Jan 2025

[26] Mercurio M, Gasparini G, Sanzo V et al (2022) Cemented total knee arthroplasty shows less blood loss but a higher rate of aseptic loosening compared with cementless fixation: an updated meta-analysis of comparative studies. J Arthroplast 37(9):1879–1887. e4 10.1016/j.arth.2022.04.013 35452802

[27] Liddle A, Pandit H, O’brien S et al (2013) Cementless fixation in Oxford unicompartmental knee replacement: a multicentre study of 1000 knees. Bone Joint J 95(2):181–7 23365026 10.1302/0301-620X.95B2.30411

[28] Whiteside LA (1995) Effect of porous-coating configuration on tibial osteolysis after total knee arthroplasty. Clin Orthop Relat Res (1976–2007) 321:92–7 7497691

[29] Kamath AF, Siddiqi A, Malkani AL, Krebs VE (2021) Cementless fixation in primary total knee arthroplasty: historical perspective to contemporary application. JAAOS-J Am Acad Orthop Surg 29(8):e363–e379 10.5435/JAAOS-D-20-00569 33399290

[30] Melton LJ (2001) The prevalence of osteoporosis: gender and racial comparison. Calcif Tissue Int 69(4):179 11730244 10.1007/s00223-001-1043-9

[31] AOANJRR Hip, Knee and Shoulder Arthroplasty 2022 Annual Report Available at: https://aoanjrr.sahmri.com/documents/10180/732916/AOA+2022+AR+Digital/f63ed890-36d0-c4b3-2e0b-7b63e2071b16. Accessed 30 Jan 2025

[32] Gao F, Henricson A, Nilsson KG (2009) Cemented versus uncemented fixation of the femoral component of the NexGen CR total knee replacement in patients younger than 60 years: a prospective randomised controlled RSA study. Knee 16(3):200–206 19097910 10.1016/j.knee.2008.11.009

[33] Chen C, Li R (2019) Cementless versus cemented total knee arthroplasty in young patients: a meta-analysis of randomized controlled trials. J Orthop Surg Res 14:1–11 31426816 10.1186/s13018-019-1293-8 PMC6700781

